# The Institutional Worker

**Published:** 1913-05-10

**Authors:** 


THE
### INSTITUTIONAL WORKER
**Being a Special Supplement to "The Hospital"**

### RULES FOR CORRESPONDENTS.

1. Every letter, on whatever subject, must be accompanied by the coupon to be cut from the back cover (inside page) of THE HOSPITAL, current issue, and must contain the name and address of the correspondent with pseudonym for publication if desired.

2. Replies by post cannot be given, save under exceptional circumstances at the Editor's discretion.

3. Letters from Approved Homes in reply to special needs published in the Bureau should state terms and full particulars, and be sent prepaid under cover to the Editor of the Bureau with name written across coupon for identification.

4. Proprietors of Homes which have not yet been entered on the List of Approved Homes, but have spare accommodation likely to suit special needs, are invited to write for an application form for registration. The fee for registration, which includes two announcements of the Home in the Bureau and other privileges, is 10s.

5. All communications to be addressed to the Editor of THE HOSPITAL, 28 Southampton Street, Strand, London, W.C., and marked "Bureau of Information."

### Home for Imbecile Boy.

THE first number of THE HOSPITAL in each quarter contains a List of Approved Homes added to the Register during the preceding three months, with full particulars of each; we do not publish a separate list. If you will state what the parents of the little boy can afford to pay, and the neighbourhood in which it is desired to obtain a Home, at the same time giving full particulars as to the nature of the case—whether helpless or not—we will insert an announcement of your needs in the columns of the Bureau.—"E. J. E."

### Nursing in Canada.

WE would recommend you to apply to the Colonial Intelligence League, 36 Tavistock Place, London, W.C. This Association has accumulated a great deal of first-hand information regarding the conditions of nursing in Canada. An interesting article upon this subject appeared on page 317 of *The Nursing Mirror* issued on February 1 last, which is well worth your perusal.—"E. T."

### Prevention of Damage to Ward Walls.

A CORRESPONDENT has drawn our attention to the fact that she has recently patented a bedstead-attachment which effectually acts as a buffer for the prevention of damage to ward walls. It is a metal and rubber contrivance, it can easily be affixed to the leg or rail of any bedstead, and inasmuch as the rubber portion is made to revolve, the bed can be pushed along the wall without any damage being done. Full information may be obtained of Whitfield's Bedstead Company, of Birmingham. — "COTTAGE HOSPITAL."

### OUR BUREAU OF INFORMATION.

### English Nursing.

PLEASE read the rules and observe them; we cannot give postal replies. You cannot obtain an adequate idea of English nursing by adopting the course you suggest. It would be better for you to take out a short course as a paying pupil at a recognised training school. Paid positions as nurses are not as a rule given in this country to any but English-trained nurses. You might possibly, as you suggest, obtain a temporary appointment at a cottage hospital, convalescent home, or private home; the only way to do this, unless you can get a private recommendation, is to answer the advertisements which appear in the Nursing Papers, such as *The Nursing Mirror*. You will find full information upon the subject of English nursing in the book "How to Become a Nurse," by Sir Henry Burdett, published by The Scientific Press, Ltd., 28 and 29 Southampton Street, Strand, London, W.C.—"U. K."

### Suitability of Persons for Out-Patient Treatment.

THE question of the suitability of persons for out-patient relief cannot be defined by a hard-and-fast wage limit, and any attempt to frame or to carry out a regulation based upon such a limit would neither be to the best interests of the hospital nor the patient. Each case must be judged on its own merits, and the true tests are: (1) whether the patient can or cannot pay for the specific treatment applied for; (2) whether or not that specific treatment is provided by some other body, to whom the patient should more properly apply. It is obvious, for instance, that the members of a large family with an income of, say, 35s. a week, and possibly reduced in circumstances through continued illness, are more eligible for hospital relief than a single man with no dependents and a weekly income of 25s. It is also equally obvious that it would be an abuse of hospital funds to deal with a case already provided for by some other agency.—"OUT-PATIENT CLERK."

### Anglo-Italian Nursing Service.

THERE are openings for English nurses, desiring experience of hospital life in Italy, in connection with the Scuola Convitto "Regina Elena," Rome. This school has been formed for the purpose of training Italian probationers on "Florence Nightingale" lines. The staff is English trained, consisting of matron, assistant matron, sisters, and sisters on probation. English nurses are first appointed as sisters on probation, and work under the English ward sister with an Italian staff nurse and Italian probationers, and if found to be suitable they are taken on as ward sisters, as vacancies occur. Sisters on probation receive 70 francs per month; sisters, 85 francs; and after two years' service, 100 francs, together with an allowance of 125 francs a year for Uniform. The engagement is for a period of two years, and may be extended. Subject to the fulfilment of the terms

### INVITATION TO READERS.

**The Editor is prepared to make known without charge the of any who may be sick or in difficulties, and to guide them in making choice of Homes for treatment or convalescence. Reduced terms can often be arranged with Homes on the Approved List for those unable to pay full fees. Queries are specially invited from those who are engaged in any kind of philanthropical work. A new edition of the leaflet describing the purposes of the Bureau of Information is now ready and will be sent free of charge on application.**

### Preparation of Catgut.

THE following method is a good one: Roll the catgut tightly on glass reels; tie the ends firmly, and drop the catgut as wound into clean glass jars. Cover catgut with solution of iodine 300 parts, and ether 1,700 parts, and leave immersed for seven days in air-tight jars. Then pour off iodine solution (do not throw away); pour ether on sutures, and continue to pour off and on until it comes away clear. The catgut is then ready for use. —"THEATRE SISTER."

### Nitrate of Silver Holder.

YOU will find it far less expensive to purchase nitrate of silver sticks without the fancy wooden handle in which they are often sold. A simple and quite efficient holder may be made by cutting off the point of a quill tooth-pick; the silver nitrate stick is then inserted into the quill, and the junction bound with a piece of adhesive plaster. —"DISPENSER."

### Sterilisation of Gloves.

SURGEONS' gloves should be sterilised in boiling water; steam sterilisation is injurious. If the surgeon requires dry gloves, powder the interior, tie the open ends of each pair with a piece of $\frac{1}{16}$-inch rubber tubing, placed in position like a tourniquet, and boil. This method sterilises the exterior of the gloves, but does not kill bacteria on the inside.—"THEATRE."

### Private Home for Lunatics.

Please read the rules and observe them—a coupon is required with each inquiry. Your question is not very well defined. There are no statutory regulations regarding the reception of slight mental cases (uncertified) at a private Home, for these cases do not come within the scope of the Lunacy Act. A licence is, however, necessary for keeping certified lunatics, and new licences are only granted in exceptional circumstances. The licensing jurisdiction is exercised by the justices of every County and Quarter Sessions Borough, with the exception of the Counties of London and Middlesex, the Cities of London and Westminster, and certain parishes with a radius of seven miles which are within the jurisdiction of the Lunacy Commissioners.—" M. D."

### Poor Relief in Australia.

Poor relief in the States of Australia, with the possible exception of Western Australia, is supplied through voluntary agencies. The Charitable Aid and Benevolent Societies for outdoor relief and the various homes for in-door relief, as well as hospitals for the sick poor, are, by way of encouragement by the State, to perform the function of public relief, allowed liberal grants towards their funds, usually on the principle of a subsidy of £1 for every £1 subscribed. In some of the States the local authorities are empowered to contribute as well. —" Traveller."

### Medico-Psychological Certificate.

Candidates for the certificate of the Medico-Psychological Association must have completed a course of three years' training at a recognised asylum for the insane; most asylums in the United Kingdom of 100 beds and upwards are so recognised. You should apply to the Medical Superintendent of one of the County Asylums, or to the Clerk of the Metropolitan Asylums Board, Embankment, London, W.C. You will find fuller particulars and a List of Institutions in the book " How to Become a Nurse," published by The Scientific Press, Ltd., 28 and 29 Southampton Street, Strand, London, W.C.—" W. L."

### Hospital for Consumption, South Coast.

You do not mention in your inquiry what form of tuberculosis the child suffers from, but if it is pulmonary tuberculosis an excellent institution on the South Coast which deals with these cases in the early stages is the Royal National Hospital for Consumption and Diseases of the Chest, Ventnor, Isle of Wight. Admission is by subscriber's letter, and a weekly payment of 10s. Apply to the Secretary, 18 Buckingham Street, Strand, London, W.C.—" M. R."

of the engagement, travelling expenses to Rome and back to England are allowed. Application should be made to the Matron, Miss D. A. Snell.—" R. E."

### Laundry Washing of Blankets.

White and grey blankets should be washed in cool water, but never ice-cold. Use four gallons soft soap (see formula) and four ounces of borax for a washer 60×32. After the suds are prepared put in the blankets, and wash for half an hour. Rinse twice, for a quarter of an hour each time, in water of the same temperature. Put the blankets through the hydro extractor, and dry, either out of doors or in the laundry. Care should be taken to avoid frost—and on no account should the blankets be hung in the drying room. *Formula for Preparing Soft Soap*: Take twenty-four pounds of potash and thirty-six of soap grease, mix in fifteen gallons of water; simmer for eighteen hours, and add enough water to make a hundred gallons.—" Laundrymaid."

### Home for Epileptic Woman.

Please read the rules. A coupon should be enclosed with each inquiry. The following Homes receive epileptic women, and are most likely to meet your requirement: The National Society for Epileptics, Chalfont Colony, Bucks, receives approved patients at a charge of 10s. a week, subject to a reduction, under special circumstances, at the discretion of the committee. Application should be made to the Secretary, Denison House, Vauxhall Bridge Road, Westminster, S.W. The Home for Epileptics, Maghull, near Liverpool, receives cases for 10s. 6d. a week; application should be made to the Secretary, 2 Exchange Street East, Liverpool. Should you be unsuccessful, we shall be pleased to make your want known in the columns of our Bureau.—" A. O. C."

### Institution Facts and Figures.

### Question for May.

During a period of four weeks in the winter months state what was the quantity of gas consumed in your institution for purposes other than illuminating, its approximate cost, the quantity of each kind of fuel used and its cost, deducting the cost of fuel for laundry purposes (if any).

In answering this question the following particulars should be given: (1) Number of wards and size of each (in beds); (2) total number of coal fires (*a*) in the wards, (*b*) in the rest of the building; (3) the number of gas-cooking and other stoves and sterilisers; (4) the name of the heating and hot-water supply service.

In the ensuing month contributors will be asked to give fuller particulars upon this subject by describing the character of the buildings, apparatus, etc.

Note.—The figures must be the result of actual observation and computation, not estimates. The best answers will be published, and for each contribution considered by the Editor to be of sufficient interest for publication payment of Five Shillings will be made.

Rules.

The following rules must be observed by all those answering the Facts and Figures Questions:—

1. Answers must be written on one side of the paper. They must bear the name and address of the sender and be accompanied by coupon to be cut from the back cover (inside page) of the current issue of The Hospital. A pseudonym must be chosen if the name is not to be published.
2. Answers must be addressed to the Editor of The Hospital, 28-29 Southampton Street, Strand, London, W.C.; they must be marked in the left-hand corner " Facts and Figures," and must be received before the end of the month.

### Begging Letters.

The London Mendicity Society, 9 Red Lion Square, London, W.C., undertakes the investigation of begging letters, and enables subscribers to give timely assistance to those really deserving of relief. Amongst other objects of this Society are the discouragement of money relief to street beggars, the distribution of food tickets to cases of distress in the streets, and the salvation of children found begging in the streets, who, when convicted, are sent by the Society to industrial schools, where they are taught a trade.—" S. B."

### How to Become a Hospital Nurse.

Please read the rules and observe them. A coupon should be enclosed with each inquiry. If you will refer to the Bureau columns of last week's issue of The Hospital, you will find a reply to your inquiry under this heading.—" F. M. T."

### Home for Chronic Case.

Please read the rules. A coupon should be enclosed with each inquiry. We cannot give postal replies. You will find a List of Approved Homes added to our Register, together with full particulars, in the first number of The Hospital commencing each quarter. Amongst those published on January 4 and April 5, 1913, you may be able to select one suitable for the case you mention. Should you be unsuccessful, we shall be pleased to make your want known through the columns of the Bureau.—" C. R. J. E."

### The Editor's Letter-Box.

### Communications have been received and attended to from:—

Nurse F. M. Young, A. M. Ogle, and M. Ward.

National Blind Relief Society (Rev. J. Pullein-Thompson).—We are glad to hear that through the interest of friends you have been able to elect Mrs. E. J. Le Mar as a pensioner of your Society, and that she will now receive the monthly pension of 10s. instead of awaiting her turn. We will make the arrangement you suggest, and advise you upon the matter at an early date.

### Editor's Notices.

**Contributions:**

Contributions should be written, or preferably typed, on one side of the paper only, and all articles sent in are accepted upon the distinct understanding that they are forwarded to THE HOSPITAL only.

The Editor cannot undertake to return MSS. not used, but when a stamped directed envelope is enclosed the MSS. may be returned if a special request is made.

Accepted articles and paragraphs of news will be paid for after publication at the scale rate.

**Address:**

To prevent delay all contributions and letters on editorial business must be addressed exclusively to the Editor, "The Hospital," 29 Southampton Street, Strand, London, W.C. It is important that this regulation shall be strictly observed.

**Correspondence:**

Correspondence on all subjects is invited. The name and address of correspondents must be given as a guarantee of good faith, but not necessarily for publication.

**Special Articles:**

Special articles are invited, and questions, inquiries, and paragraphs upon all matters relating to the work, administration, and management of general, special, mental, fever, cottage and convalescent hospitals, sanatoria, homes, institutions, societies and organisations for the treatment or care of the sick, injured and dependants of all classes. Every matter affecting the interests and welfare of the staff of all grades working in these institutions will receive special consideration.

**Administrative Medicine, Research and Items of News:**

Special terms will be paid for approved articles on subjects in this wide field by experts who have made some section of it their special study and interest. We wish to give prominence to every movement tending to advance accuracy of diagnosis, efficiency in treatment, and the development of modern methods to eradicate disease and promote the welfare of the suffering.

**A Bureau of Information:**

We invite inquiries and applications for information and help in providing for that numerous class of sufferers who require special care or house-room which they cannot obtain in their own homes or secure for themselves. We cannot, however, prescribe, or recommend practitioners.

**Books for Review:**

Publishers are particularly requested to send advance proofs of any new books of importance whenever possible, as the Editor has made arrangements to publish immediate reviews on a new plan.

**Photographs, Plans, Blocks, and Illustrations:**

It is requested that wherever possible MSS. may be accompanied by illustrations in any of the above forms. The name of the sender and of the article to which it belongs should be written on the back of each photograph, drawing, or block for purposes of identification.

**Local Papers:**

Newspapers containing reports or news paragraphs should be marked and addressed to the Sub-Editor.

**The Coupon System:**

A coupon will be found at the bottom of the third inside page of the cover each week. This coupon must be attached to every question or inquiry to which an answer is desired in THE HOSPITAL.

### To Fill that Vacancy.

As a medium for announcing vacancies in every department of Institutional Life, the exceptional value of *The Institutional Worker* is evidenced by the large number of such notices that appear week by week in this section of THE HOSPITAL. No more adequate proof of the usefulness of *The Institutional Worker* can be desired than the fact that the Responsible Officers of the most important Institutions in the United Kingdom employ its columns again and again whenever they have a vacancy to fill upon their staffs. It also provides exceptional facilities for every class of advertisement affecting Institutional Life and Work, and as a means of securing re-engagements in any section of the Institutional, Health, and Sanitary Services it is absolutely incomparable, since no other journal possesses such a wide and comprehensive circulation in these directions as THE HOSPITAL. If you are looking for a post an announcement of twenty-one words, costing the small sum of 1s., may save you many hours of anxious thought, and will certainly bring your requirement immediately before those most likely to need your services.

This form may be used for Appointments Wanted advertisements, and when filled up should be posted to
*The Manager*, THE HOSPITAL,
28 and 29 Southampton Street,
Strand, W.C.

Appointments Wanted, 21 words ... 1s. 0d.

| | | |
|---|---|---|
| | | |
| | | |
| | | |
| | | |
| | | |
| | | |
| | | |

### Manager's Notices.

**Letters relating to the publication, sale, and advertising departments of "The Hospital" must be addressed to The Manager, "The Hospital," The Hospital Building, 28 and 29 Southampton Street, London, W.C.**

**Subscriptions** may begin at any time, and are payable in advance. Cheques and Post-Office Orders should be crossed London County and Westminster Bank, Covent Garden Branch, and made payable to the Manager of THE HOSPITAL.

RATES OF SUBSCRIPTION (including Postage).

| *United Kingdom.* | | *Foreign and Colonial.* | |
|---|---|---|---|
| Three Months ... ... | 2s. 0d. | Three Months ... ... | 2s. 6d. |
| Six Months ... ... | 4s 0d | Six Months ... ... | 5s. 0d. |
| Twelve Months ... ... | 6s. 6d. | Twelve Months ... ... | 8s. 8d. |

**Advertisements:**

To ensure insertion, all advertisements for THE HOSPITAL must reach the Manager not later than Wednesday morning in each week. For scale of charges see pages vii and 4.

**Remittances:**

**It is especially requested that remittances be made by Cheque or Postal Order, and in halfpenny stamps only when the amount is under sixpence.**

LATEST EDITION

OF

THE NURSE'S PRONOUNCING DICTIONARY

OF

MEDICAL TERMS AND NURSING TREATMENT

Compiled by

HONNOR MORTEN

Seventh Edition.

Revised and Edited by

Mary I. Burdett.

BECAUSE:

IT IS QUITE UP TO DATE.

CONTAINS NO OBSOLETE TERMS.

IS ABSOLUTELY DEPENDABLE.

The proofs were passed by a highly Qualified Medical Man, consequently the utmost reliance can be placed in the information within its pages.

GET A COPY TO-DAY.

Of all Booksellers, or direct from THE PUBLISHERS,

THE SCIENTIFIC PRESS, Ltd.,
28 & 29 Southampton Street,
Strand, London, W.C.

